# Biclustering of microarray data with MOSPO based on crowding distance

**DOI:** 10.1186/1471-2105-10-S4-S9

**Published:** 2009-04-29

**Authors:** Junwan Liu, Zhoujun Li, Xiaohua Hu, Yiming Chen

**Affiliations:** 1 School of Computer, National University of Deference Technology, Changsha, PR China; 2 School of Computer Science, Central South University of Forestry and Technology, 498 Shaoshan South Road, Changsha, PR China; 3 School of Computer Science & Engineering, Beihang University, 37 Xueyuan Road, Haidian District, Beijing, PR China; 4 College of Information Science and Technology, Drexel University, Philadelphia, USA; 5 School of Information Science and Technology, Hunan Agricultural University, Furong District, Changsha, PR China

## Abstract

**Background:**

High-throughput microarray technologies have generated and accumulated massive amounts of gene expression datasets that contain expression levels of thousands of genes under hundreds of different experimental conditions. The microarray datasets are usually presented in 2D matrices, where rows represent genes and columns represent experimental conditions. The analysis of such datasets can discover local structures composed by sets of genes that show coherent expression patterns under subsets of experimental conditions. It leads to the development of sophisticated algorithms capable of extracting novel and useful knowledge from a biomedical point of view. In the medical domain, these patterns are useful for understanding various diseases, and aid in more accurate diagnosis, prognosis, treatment planning, as well as drug discovery.

**Results:**

In this work we present the CMOPSOB (Crowding distance based Multi-objective Particle Swarm Optimization Biclustering), a novel clustering approach for microarray datasets to cluster genes and conditions highly related in sub-portions of the microarray data. The objective of biclustering is to find sub-matrices, i.e. maximal subgroups of genes and subgroups of conditions where the genes exhibit highly correlated activities over a subset of conditions. Since these objectives are mutually conflicting, they become suitable candidates for multi-objective modelling. Our approach CMOPSOB is based on a heuristic search technique, multi-objective particle swarm optimization, which simulates the movements of a flock of birds which aim to find food. In the meantime, the nearest neighbour search strategies based on crowding distance and ϵ-dominance can rapidly converge to the Pareto front and guarantee diversity of solutions. We compare the potential of this methodology with other biclustering algorithms by analyzing two common and public datasets of gene expression profiles. In all cases our method can find localized structures related to sets of genes that show consistent expression patterns across subsets of experimental conditions. The mined patterns present a significant biological relevance in terms of related biological processes, components and molecular functions in a species-independent manner.

**Conclusion:**

The proposed CMOPSOB algorithm is successfully applied to biclustering of microarray dataset. It achieves a good diversity in the obtained Pareto front, and rapid convergence. Therefore, it is a useful tool to analyze large microarray datasets.

## Background

With the advent of DNA microarray technology, it is allowable for simultaneously measuring the expression level of thousands of genes under different conditions in a single experiment. In this way the scientific community can collect a huge amount of gene expression datasets. In recent decade years, microarray technique has been widely used in several contexts such as tumour profiling, drug discovery and temporal analysis of cell behaviour [1,2]. Applications of these microarray data contain the study of gene expression in yeast under different environmental stress conditions and the comparisons of gene expression profiles for tumour from cancer patients. In addition to the enormous scientific potential of DNA microarrays to help in understanding gene regulation and interactions, microarrays have important applications in pharmaceutical and clinical research [1]. By comparing gene expression in normal and disease sells, microarrays may be used to identify disease genes and targets for therapeutic drugs.

Mining patterns from those microarray dataset is an important research problem in bioinformatics and clinical research. These patterns relate to disease diagnosis, drug discovery, protein network analysis, gene regulate, as well as function prediction.

Clustering techniques have been widely applied in gene expression analysis. It can identify set of genes with similar profiles. However, clustering methods assume that related genes have the similar expression patterns across all conditions, which is not reasonable especially when the dataset contains many heterogeneous conditions. These algorithms such as k-means [2], hierarchical clustering [3], self organizing maps [4] work in the full dimension space, which consider the value of each point in all the dimensions and try to group the similar points together. However, relevant genes are not necessarily related to every condition, so fail to group subset of genes that have similar expression over some but not all conditions. So biclustering is proposed for grouping simultaneously genes set and condition set over which the gene subset exhibit similar expression patterns. Cheng and Church [5] introduce first biclustering to mine genes clusters with respect to a subset of the conditions from microarray data. Up to date, a number of biclustering algorithms for microarray data analysis have been developed such as δ-biclustering [5], pClustering [6], statistical-algorithmic method for biclustering analysis (SAMBA) [7], spectral biclustering [8], Gibbs sampling biclustering [9], simulated annealing biclustering [10], etc. See [11] for a good survey.

Among the various clustering approaches, many methods are based on local search to generate suboptimal solutions. In recent years heuristics optimization has become a very popular research topic. To order to escape from local minima, many evolutionary algorithms (EA) have been proposed in [12-14] to discover global optimal solutions in gene expression data. These methods apply single-objective EA to find optimal solutions. If a single objective is optimized, the global optimum solution can be found. But in the real-world optimization problem, there are several objectives in conflict with each other to be optimized. These problems with two or more objective functions are called multi-objective optimal problem and require different mathematical and algorithmic tools to solve it. MOEA generates a set of Pareto-optimal solutions [12] which is suitable to optimize two or more conflicting objectives such as NSGA-II [13], PAES [14] and SPEA2 [15].

When mining biclusters from microarray data, we must optimize simultaneously several objectives in conflict with each other, for example, the size and the homogeneity of the clusters. In this case multi-objective evolutionary algorithms (MOEAs) are proposed to discover efficiently global optimal solution. Among many MOEA proposed the relaxed forms of Pareto dominance has become a popular mechanism to regulate convergence of an MOEA, to encourage more exploration and to provide more diversity. Among these mechanisms, ϵ-dominance has become increasingly popular [16], because of its effectiveness and its sound theoretical foundation. ϵ-dominance can control the granularity of the approximation of the Pareto front obtained to accelerate convergence and guarantee optimal distribution of solutions. At present, several algorithms [17,18] adopt MOEAs to discover biclusters from microarray data.

Recently particle swarm optimization (PSO) proposed by Kebnnedy and Eberhart [19,20] is a heuristics-based optimization approach simulating the movements of a bird flock finding food. The most attractive of PSO is that there are very few parameters to adjust. So it has been successfully used for both continuous nonlinear and discrete binary single-objective optimization. With the rapid convergence and relative simplicity, PSO becomes very suitable to solve multi-objective optimization named as multi-objective PSO (MOPSO). In recent years many multi-objective PSO (MOPSO) approaches such as [21,22] has proposed. The strategy of ϵ-dominance and crowding distance [13] are introduced into MOPSO speeding up the convergence and attaining good diversity of solutions [23-28]. There are currently over twenty five different proposals of MOPSOs presented in [29]. We propose the algorithm MOPSOB [30] to mine biclusters.

In this paper, we modify the fully connected flight model and incorporate ϵ-dominance strategies and crowding distance into MOPSO, and propose a novel MOPSO biclustering framework to find one or more significant biclusters of maximum size from microarray data. Three objectives, the size, homogeneity and row variance of biclusters, are satisfied simultaneously by applying three fitness function in optimization framework. A low mean squared residue (MSR) score of biclusters denotes that the expression levels of each gene within the biclusters are similar over the range of conditions. Using the row variance as fitness function can guarantee that the found biclusters capture the subset of genes exhibiting fluctuating yet coherent trends under subset of conditions, therefore reject trivial biclusters. Therefore, we focus on finding biclusters of maximum size, with mean squared residue lower than a given δ, with a relatively high gene-dimension variance.

## Results

To determine whether the proposed methodology is able to mining better biclusters from microarray data, we have used two common gene expression datasets. In the next sections we describe an overview of the methodology and the detailed results of its application to the analysis of two real datasets.

### CMOPSOB algorithm

In this paper, we incorporate ϵ-dominance, crowding distance and the nearest neighbour search approach into MOPSO framework, and propose CMOPSOB algorithm to mine biclusters from the microarray datasets. In the solution space, after the initialization of the particle swarm, each particle keeps track of its position which is associated with the best solution achieved so far. The personal best solution of a particle is denoted by *pbest *and the best neighbour of a particle by *nbest*. The global optimal solution of the particle swarm is the best location obtained so far by any particle in the population and is named as *gbest*. The proposed algorithm consists of iteratively changing the velocity of each particle toward its *pbest*, *nbest *and *gbest *positions. The external archive (denoted as A) records non-dominated set of the particle swarm (PS) that is the final optimal solution set. Our algorithm is given in the following different steps.

### Initialization of the algorithm

We implement the search of optimal solutions in a discrete binary space inspired by [20]. The value of a particle on each dimension (e.g. *x*_*id *_presents the value of particle *i *on dimension *d*) is only set to zero or one. We define the velocity of particle as the probability which a binary bit changes. For example each *v*_*id *_represents the probability of bit *x*_*id *_being the value 1. Therefore *v*_*id *_must be assigned to the interval [0, 1]. Personal best position of each particle *i *found so far is maintains in *pbest*_*i *_whose value of each dimension *d *(*pbest*_*id*_) is integers in {0, 1}. Initialization process first initializes the location and velocity of each particle, and then external archive is initialized. Lastly we initialize global bests (*gbest*) of each particle.

**Step-1 **Initialize the particle swarm (PS) with size S

The particle swarm is initialized with a population of random solutions

For *i *= 1 to *S *

   For *d *= 1 to *N *(the number of dimension)

      Initialized *x*_*id *_and *v*_*id *_randomly

   Endfor

   Evaluate the *i*-th particle *x*_*i*_

   *pbest*_*i *_= *x*_*i *_(the personal bests for *x*_*i *_is initialized to be the original position)

   *nbest*_*i *_= *x*_*i *_(the best neighbours of *x*_*i *_is initialized to be the original position)

Endfor

**Step-2 **Initialize external archive and the global bests (*gbest*) of each particle

Non-dominated set of initialized PS is constructed depending on ϵ-dominance relation, which is reserved in the external archive (A). Then global bests (*gbest*) for each particle in the PS is selected randomly from A. Lastly, the crowding distance of each particle in A is computed.

### Iterative update operation of the algorithm

Each iteration consists of the following three processes. The first is evaluation of each particle. Secondly, the velocity *v*_*id *_of each particle is updated based on particle *i*'s best previous position (*pbest*_*id*_), the best neighbour of particle *i *and the best previous position of all particle (*gbest*_*id*_). Lastly each particle flies its new best position, and global bests of each particle and external archive is updated.

**Step-3 **Update velocity and location of each particle

In discrete search space, a particle may fly to nearer and farther position of the hypercube by changing various numbers of bits. Thus update process of the particle is implemented to generate new swarm as the following rule.

(1) $$\begin{array}{lll} v_{id} & = & w*v_{id}+c_{1}r_{1}*(pbest_{id}-x_{id})\\ & & +c_{2}r_{2}*(nbest_{id}-x_{id})+c_{3}r_{3}*(gbest_{id}-x_{id}) \end{array}$$

(2) $$\begin{array}{l} if(rand()<S(v_{id}))\;then\;x_{id}=1;\\ \begin{array}{cc} else & x_{id}=0 \end{array} \end{array}$$

Where *c*_1_, *c*_2 _and *c*_3 _are three constants which are used to bias the influence between *pbest*, *nbest *and *gbest*, and we assume *c*_1 _= *c*_2 _= *c*_3 _= 1. The parameter *w *is inertia weight, and we set *w *= 0.5. Two parameters *r*_1_, *r*_2 _and *r*_3 _are random numbers in the range [0, 1]. The parameter *pbest*_*id*_, *nbest*_*id *_and *gbest*_*id *_are integers in {0, 1}, and *v*_*id *_(as a probability) must be constrained to the interval [0, 1]. The function S (v) is a logistic transformation and rand () is a quasi-random number selected from a uniform distribution in [0, 1].

**Step-4 **Evaluate and update each particle in PS

Each particle in PS has a new location, if the current location is dominated by its personal best, then the previous location is kept, otherwise, the current location is set as the personal best location. If they are mutually non-dominated, we select the location with least crowding distance.

**Step-5 **Compute crowding distance and update external archive

Based on ϵ-dominance relation, the non-dominated set of PS is constructed and combined into the current external archive and then get a bigger set *leader*. After computing crowding distance in *leaders*, a new external archive is got by selecting the S particles with least crowding distance.

**Step-6 **Update global bests of each particle

Update the global bests of each particle in PS that are selected randomly from A which mainly aim at searching in whole space for global optimization solutions.

The algorithm iteratively updates position of the particle until user-defined number of generations are generated and lastly converges to the optimal solution, or else, implements iteration go to step-3.

**Step-7 **Return the set of biclusters

The particles in external archive A are the optimal solutions that present the set of biclusters.

### Testing

Our algorithm CMOPSOB is implemented on two well-known datasets, yeast and human B-cells expression datasets. To compare the performance of the proposed algorithm with MOEA [17] and MOPSOB [30], a criteria the coverage is defined as the total number of cells in microarray data matrices covered by the found biclusters.

### Yeast dataset

Table 1 shows the information of ten biclusters out of the one hundred biclusters found on the yeast dataset. The one hundred biclusters cover 75.2% of the genes, 100% of the conditions and in total 53.8% cells of the expression matrix, while Ref [17] and Ref [30] report an average coverage of 51.34% and 52.4% cells respectively.

**Table 1:** Information of biclusters found on yeast dataset.

Bicluster	Genes	Conditions	Residue	Row Variance
1	79	17	205.44	711.08
8	101	16	221.12	685.33
12	621	11	200.11	1634.32
21	1156	10	221.42	1385.08
32	543	12	199.11	986.09
44	325	15	231.04	999.55
53	1215	13	281.82	778.73
69	87	16	209.33	1085.22
81	1224	8	201.77	943.45
88	1022	9	203.89	911.75

This table shows the number of genes and conditions, the mean squared residue and the row variance of ten biclusters out of the one hundred biclusters found on the yeast dataset.

**Figure 1:**
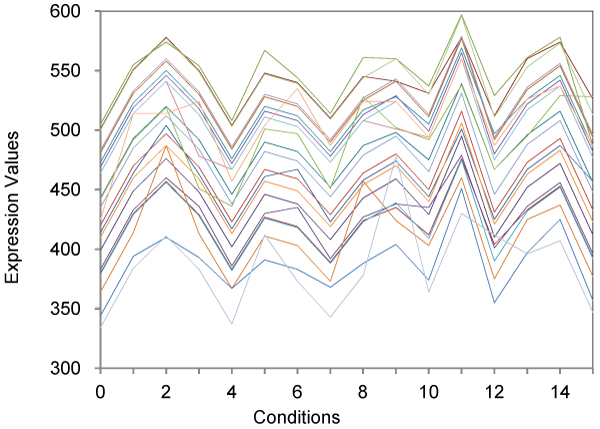
**Small biclusters of size 24 × 15 on the yeast dataset**. This figure shows the expression value of 24 genes under 15 conditions from the found biclusters.

Figure 1 depicts sample gene expression profiles for small biclusters (bicluster 69) for the yeast dataset. It shows that 24 genes present a similar behaviour across 15 experimental conditions.

### Human B-cells expression dataset

Table 2 shows the information of ten biclusters out of the one hundred found on the human dataset. The one hundred biclusters found on the human dataset cover 38.3% cells of dataset(48.1%of the genes and 100% of the conditions), whereas an average of 20.96% and 36.9% cells are covered in [17] and [30], respectively.

**Table 2:** Biclusters found on human dataset.

Bicluster	Genes	Conditions	Residue	Row Variance
1	1088	27	895.25	3141.25
11	812	39	774.26	2598.36
14	1024	32	986.74	3698.54
21	997	38	1024.11	3014.22
29	741	43	1078.95	2987.84
39	135	79	1098.76	3012.88
48	919	41	980.66	3111.54
54	841	72	1125.87	3987.65
69	298	79	986.58	3897.64
91	871	43	788.19	7843.98

This table shows the number of genes and conditions, the mean squared residue and the row variance of ten biclusters out of the one hundred biclusters found on the human dataset.

### Comparative analysis

Among the different MOEAs algorithms, NSGA2 are the best multi-object optimization algorithm. Mitra and Banka [17] incorporates NSGA2 with local search strategies to solve biclustering problem denoted as NSGA2B(NSGA2 biclustering).

In this section, we compare the proposed CMOPSOB with two well known MOEA biclustering and MOPSOB [30] on the yeast data and the human B-cells expression data. Parameters are set to same as MOPSOB [30]. We use a crossover probability of 0.75 and a mutation probability of 0.03 for NSGA2B. For MOPSOB and CMOPSOB, we set ε = 0.01. Comparative analysis of three algorithms is shown in Table 3.

**Table 3:** Comparative study of three algorithms.

	NSGA2B		MOPSOB		CMOPSOB	
Dataset	Yeast	Human	Yeast	Human	Yeast	Human
Avg. MSR	234.87	987.56	218.54	927.47	208.86	921.66
Avg. size	10301.71	33463.70	10510.8	34012.24	11085.44	36400.58
Avg. genes	1095.43	915.81	1102.84	902.41	1118.41	931.11
Avg. conditions	9.29	36.54	9.31	40.12	9.45	40.14
Max size	14828	37560	15613	37666	15795	37679

This table compares the performance of three algorithms, and gives the average of mean squared residue, the average number of genes and conditions, the average size and maximal size of the found biclusters

Table 3 shows that the biclusters found by CMOPSOB is characterized by a slightly lower squared residue and a higher bicluster size than those by NSGA2B and MOPSOB on both yeast dataset and human dataset. However when comparing MOPSOB with NSGA2B, we find the biclusters found by MOPSOB have better quality than those found by NSGA2B.

In total it is clear from the above results that the proposed CMOPSOB algorithm performs best in maintaining diversity, achieving convergence.

### Analysis of biological annotation enrichment of gene clusters

The gene ontology (GO) project  provides three structured, controlled vocabularies that describe gene products in terms of their associated biological processes, cellular components and molecular functions in a species-independent manner. The enrichment of functional annotations in genes contained in biclusters is evaluated using Onto-Express tool [31]. To determine the biological relevance of the biclusters found by CMOPSOB on the yeast dataset in terms of the statistically significant GO annotation database, we feed genes in each bicluster to Onto-Express  and obtain a hierarchy of functional annotations in terms of Gene Ontology for each bicluster. Here only categories with p-values less than 0.01 were considered statistically significant.

The degree of enrichment is measured by p-values which use a cumulative hyper-geometric distribution to compute the probability of observing the number of genes from a particular GO category (function, process and component) within each bicluster. For example, the probability *p *for finding at least *k *genes from a particular category within a bicluster of size *n *is given in (3).

(3) $$p=1-\sum_{i=0}^{k-1}\frac{\left(\begin{array}{c} m\\ i \end{array}\right)\left(\begin{array}{c} g-m\\ n-i \end{array}\right)}{\left(\begin{array}{c} g\\ n \end{array}\right)}$$

Where *m *is the total number of genes within a category and *g *is the total number of genes within the genome [32]. The p-values are calculated for each functional category in each bicluster to denote how well those genes match with the corresponding GO category.

Table 4 lists the significant shared GO terms (or parent of GO terms) used to describe the set of genes in each bicluster for the process, function and component ontology. For example for cluster C_16_, we find that the genes are mainly involved in lipid transport. The tuple (n = 23, p = 0.00013) means that out of 96 genes in cluster C_16_, 23 genes belong to lipid transport process, and the statistical significance is given by the p-value of 0.00013. Those results mean that the proposed CMOPSO biclustering approach can find biologically meaningful clusters.

**Table 4:** Significant GO terms of genes in clusters.

Cluster No.	No. of genes	Process	Function	Component
16	96	Lipid transport (n = 23, p = 0.00013)	Oxidoreductase activity (n = 12, p = 0.00376)	membrane (n = 18, p = 0.0064)
56	141	Cell organization and biogenesis (n = 31, p = 0.0046)	Protein transporter activity (n = 5, p = 0.0035)	Nucleus (n = 25, p = 0.0043)
81	1024	Cellular process (n = 37, p = 0.0023)	tRNA methyltransferase activity (n = 14, p = 0.0012)	Cytosolic small ribosomal subunit (n = 11, p = 0.0065)

This table lists the significant shared GO terms which are used to describe genes in each bicluster for the process, function and component ontology. Here, only shows the terms whose p-values are smaller than 0.01.

## Discussion

Although there is the amount of information in large and diverse databases of genome-wide expression profiles, mining of meaningful biological knowledge from those datasets remains enormous challenges. Multi-objective evolutionary biclustering is a global search heuristic approach, and demonstrate better performance as compared to existing various greedy biclustering methods proposed in the literature [17]. But this approach need spend too much computation time in order to achieve better convergence and diversity. PSO is initialized with a population of random solutions which the point is same as genetic algorithm (GA). The difference is, however, each potential solution of PSO named as particle is also assigned a randomized velocity, and then flown to the optimal solution in the solution space. Another important difference is the fact that PSO allows individuals to benefit from their past experiences whereas in an evolutionary algorithm, normally the current population is only retained solution of the individuals. This paper introduces a new global search framework for biclustering based on MOPSO approach. Because PSO method does not use the filtering operation such as crossover and mutation and the whole swarm population maintains constant during the search process. So in addition to attaining better convergence and diversity, our approach proposed here offers great advantages over evolutionary methods of biclustering. Our method can also speed up the process of search. In the future, we will adapt various types of biological methods such as immune system to mining biclusters from microarray datasets. At the same time, we will combine the advantage of those evolutionary computations to propose hybrid biclustering methods for biclustering of microarray dataset.

## Conclusion

In this work, we have provided a novel multi-objective PSO framework for mining biclusters from microarray datasets. We focus on finding maximum biclusters with lower mean squared residue and higher row variance. Those three objectives are incorporated into the framework with three fitness functions. We apply MOPSO to quicken convergence of the algorithm, and ϵ-dominance and crowding distance update strategy to improve the diversity of the solutions. The results on the yeast microarray dataset and the human B-cells expression dataset verify the good quality of the found biclusters, and comparative analysis show that the proposed CMOPSOB is superior to NSGA2B and MOPSOB in terms of diversity, convergence.

## Methods

### Biclusters

Given a gene expression data matrix D = G × C = {*d*_*ij*_} (here *i *∈ [1, *n*], *j *∈ [1, *m*]) is a real-valued *n *× *m *matrix, here G is a set of *n *genes **{g_1_, g_2_, ⋯, g_n_}**, C a set of m biological conditions **{c_1_, c_2_, ⋯, c_n_}**. Entry *d*_*ij *_means the expression level of gene *g*_*i *_under condition *c*_*j*_.

#### Definition 1 Bicluster

Given a gene expression dataset D = G × C = {*d*_*ij*_}, if there is a submatrix B = *g *× *c*, where *g *⊂ G, *c *⊂ C, to satisfy certain homogeneity and minimal size of the cluster, we say that B is a bicluster.

#### Definition 2 Maximal bicluster

A bicluster B = *g *× *c *is maximal if there exists not any other biclusters B' = *g*' × *c*' such that *g*' ⊂ G and *c*' ⊂ C,

#### Definition 3 Dimension mean

Given a bicluster B = *g *× *c*, with subset of genes *g *⊂ G, subset of conditions *c *⊂ C, *d*_*ij *_is the value of gene *g*_*i *_under condition c_*j *_in the dataset D. We denote by *d*_*ic *_the mean of the ith gene in B, *d*_*gj *_the mean of the jth condition in B. We also denote by *d*_*gc *_the mean of all entries in B. These values are defined as follows, where Size (g, c) = |g||c| presents the size of bicluster B.

(4) $$d_{ic}=\frac{1}{\left|c\right|}\sum_{j\in c}d_{ij}$$

(5) $$d_{gj}=\frac{1}{\left|g\right|}\sum_{i\in g}d_{ij}$$

(6) $$d_{gc}=\frac{1}{\left|g||c\right|}\sum_{i\in g,j\in c}d_{ij}$$

#### Definition 4 Residue and mean square residue

Given a bicluster B = *g *× *c*, to assess the difference between the actual value of an element *d*_*ij *_and its expected value, we define by r(*d*_*ij*_) the residue of *d*_*ij *_in bicluster B in (7). Therefore the mean squared residue (MSR) of B is defined as the sum of the squared residues to assess overall quality of a bicluster B in (8).

(7) *r*(*d*_*ij*_) = *d*_*ij *_- *d*_*ic *_-*d*_*gi *_+ *d*_*gc*_

(8) $$MSR(g,c)=\frac{1}{\left|g||c\right|}\sum_{i\in g,j\in c}r{\left(d_{ij}\right)}^{2}$$

#### Definition 5 Row variance

Given a bicluster B = *g *× *c*, the ith gene variance in B is defined by RVAR (*i*, *c*) and the overall gene-dimensional variance is defined as the sum of all genes variance as follows.

(9) $$RVAR(g,c)=\frac{1}{\left|g||c\right|}\sum_{i\in g,j\in c}{\left(d_{ij}-d_{ic}\right)}^{2}$$

(10) $$RVAR(i,c)=\frac{1}{\left|c\right|}\sum_{j\in c}{\left(d_{ij}-d_{ic}\right)}^{2}$$

Our target is mining good quality biclusters of maximum size, with mean square residue (MSR) smaller than a user-defined threshold δ > 0, which presents the maximum allowable dissimilarity within the biclusters, and with a greater row variance.

### Bicluster encoding

Each bicluster is encoded as a particle of the population. Each particle is represented by a binary string of fixed length *n*+*m*, where n and m are the number of genes and conditions of the microarray dataset, respectively. The first *n *bits are associated to *n *genes, the following *m *bits to *m *conditions. If a bit is set to 1, it means that the responding gene or condition belongs to the encoded bicluster; otherwise it does not. This encoding presents the advantage of having a fixed size, thus using simply of standard variation operations [33]. For example a biclusters consists of 16 bits (the first 8 bits corresponding to 8 genes and the following 8 bits to 8 conditions). Therefore, the following binary string:

10100010|10100110

presents the individual encoding a bicluster with 3 genes and 4 conditions, and then its size is 3 × 4 = 12. Where | is a symbol used to delimit the bits relative to the rows from the bits relative to the columns.

### Nearest neighbour flight

Most MOPSO algorithms adopt the fully connected flight model to propel the swarm particles towards the Pareto optimal front. Zhang et al. [34] includes the lattice model to escape the local optimal. This paper introduces nearest neighbour flight model. Two particles are nearest neighbours if and only if the binary encodes of two particles are only different in one bit. That is to say, two biclusters is only different in a gene or a condition. During search process, each particle searches and flies to the best position (named as *nbest*) of its nearest neighbours.

### ϵ-dominance

In this section we define relevant concepts of dominance and Pareto sets. The algorithms presented in this paper assume that all objectives are to be minimized. Objective vectors are compared according to the dominance relation defined below.

#### Definition 6 Dominance relation

Let f, g ∈ R^m^. Then f is said to dominate g (denoted as f ≻ g), iff

**(i) **∀ *i *∈ {1, ...., *m*}: f_i _≤ g_i_

**(ii) **∃ *j *∈ {1, ...., *m*}: f_j _< g_j_

#### Definition 7 Pareto set

Let F ∈ *R *^*m *^be a set of vectors. Then the Pareto set F* of *F *is defined as follows:

F* contains all vectors g ∈ F which are not dominated by any vector f ∈ F, i.e.

(11) *F *: = {g ∈ F | ∄ f ∈ F : f ≻ g}

Vectors in F* are called Pareto vectors of F. The set of all Pareto sets of F is denoted as P*(F).

#### Definition 8 ϵ-dominance

Let f, g ∈ *R*^*m*^. Then f is said to ϵ-dominate g for some ϵ > 0, denoted as f ≻_ϵ _g, iff for all *i *∈ {1, ...., *m *}

(12) (1 + ϵ) *f*_*i *_≤ *g*_*i*_.

#### Definition 9 ϵ-approximate Pareto set

Let F ⊇ *R*^*m *^be a set of vectors and ϵ > 0. Then a set *F*_ϵ _is called a ϵ-approximate Pareto set of F, if any vector g ∈ F is ϵ-dominated by at least one vector f ∈ *F*_ϵ_, i.e.

(13) ∀ g ∈ F : ∃ f ∈ *F*_ϵ _such that f ≻_ϵ _g

The set of all ϵ-approximate Pareto sets of F is denoted as P_ϵ _(F).

#### Definition 10 ϵ-Pareto set

Let *F *⊆ *R*^*m *^be a set of vectors and ϵ > 0. Then a set $F_{\epsilon }^{*}$ ⊆ *F *is called an ϵ-Pareto set of *F if*

(i) $F_{\epsilon }^{*}$ is an ϵ-approximate Pareto set of F, i.e. $F_{\epsilon }^{*}$ ∈ *P*_ϵ _*(F)*, and

(ii) $F_{\epsilon }^{*}$ contains Pareto points of X only, i.e. $F_{\epsilon }^{*}$∈ F*

The set of all ϵ-Pareto set of *F *is denoted as $P_{\epsilon }^{*}$ (*F*).

### Crowding distance

The crowding distance of a particles can estimate the density of particles including the particle [13]. The computation of the crowding distance of particle *i *is reached by estimating the size of the largest cube surrounding particle *i *without including any other particle [25]. The calculation of crowding distance of each particle is achieved as the following steps.

**Step 1 **Calculate the number of non-dominated particles in external archive A, *s *= |A|.

**Step 2 **Initialize the distance of each particle *i *to zero, A [i].distance = 0.

**Step 3 **Computes the distance of each particle. For each objective m, the following steps are implemented.

**Step 3.1 **sorting A [] in ascending objective m function values of each particle.

**Step 3.2 **Set the maximum distance to the boundary points so that they are always selected A [1].distance = A [s].distance= maximum distance.

**Step 3.3 **The distance of all other particles *i *= 2 to *s*-1 are the average distance of its two neighbouring solutions computed as follows:

A [i].distance = A [i].distance + (A [i+1].m - A [i-1].m)

Here A [i-1].m refers to the m-th objective function value of the i-th individual in the set A.

### Fitness function

Our hope is mining biclusters with low mean squared residue, with high volume and gene-dimensional variance, and those three objectives in conflict with each other are well suited for multi-objective to model. To achieve these aims, we use the following fitness functions.

(14) $$f_{1}(x)=\frac{\left|G||C\right|}{size(x)}$$

(15) $$f_{2}(x)=\frac{MSR(x)}{\delta }$$

(16) $$f_{3}(x)=\frac{1}{RVAR(x)}$$

Where G and C are the total number of genes and conditions in microarray datasets respectively. Size(x), MSR(x) and RVAR(x) denotes the size, mean squared residue and row variance of bicluster encoded by the particle × respectively. δ is the user-defined threshold for the maximum acceptable mean squared residue. Our algorithm minimizes those three fitness functions.

### Update of ϵ-Pareto set of the particle

In order to guarantee the convergence and maintain diversity in the population at the same time, we implement updating of ϵ-Pareto set of the population during selection operation similar to [16]. The following steps conclude a general scheme of the updating algorithm.

**Step 1**. For each particle *x *in the swarm X, the set Y contains particles *x' *which meet the condition that ln*x *dominate ln*x'*. Here function *f *= ln(*x*) is computed as *y*_*i *_= ⌊ln *x*_*i*_/(1 + ∈)⌋ which *x*_*i *_presents the value of the solution × in objective *i*, and *y *denotes an *m *× 1 vector.

**Step 2**. From the particle swarm X, exclude Y and those particles *x' *which meet (i) lnx' = lnx, (ii) *x *dominate *x'*.

### Datasets and data preprocessing

We apply the proposed multi-objective PSO biclustering algorithm to mine biclusters from two well known datasets. The first dataset is the yeast Saccharomyces cerevisiae cell cycle expression data [35], and the second dataset is the human B-cells expression data [36].

#### Yeast dataset

The yeast dataset collects expression level of 2,884 genes under 17 conditions. All entries are integers lying in the range of 0–600. Out of the yeast dataset there are 34 missing values. The 34 missing values are replaced by random number between 0 and 800, as in [5].

#### Human B-cells expression dataset

The human B-cells expression dataset is collection of 4,026 genes and 96 conditions, with 12.3% missing values, lying in the range of integers -750-650. Like in [5], the missing values are replaced by random numbers between -800-800. However, those random values affect the discovery of biclusters [37]. For providing a fair comparison with existing methods we set the same parameter for δ as [5], i.e., for the yeast data δ = 300, for the human B-cells expression data δ = 1200. The two gene expression dataset are taken from [5].

## Competing interests

The authors declare that they have no competing interests.

## Authors' contributions

JL proposed to use MOPSO methods to mining biclusters from gene expression data and drafted the manuscript. ZL and XH were involved in study design and coordination and revised the manuscript. YC conducted the algorithm design.
